# Advancements in Nanoparticle‐Based Therapies for Breast Cancer: Current Trends and Future Prospects

**DOI:** 10.1155/ijbc/1986406

**Published:** 2026-07-16

**Authors:** Sina M. Matalqah, Laila Mahmoud Matalqah, Abdel Rahman Al Tawaha, Arun Karnwal, Lujain Radaideh, Asem Mhaidat, Natalia Nesterova

**Affiliations:** ^1^ Department of Pharmaceutics and Pharmaceutical Technology, Faculty of Pharmacy, Pharmacological and Diagnostic Research Centre (PDRC), Al-Ahliyya Amman University, Amman, Jordan, ammanu.edu.jo; ^2^ Department of Clinical Sciences, College of Pharmacy and Health Sciences (COPHS), Ajman University, Ajman, United Arab Emirates, ajman.ac.ae; ^3^ Department of Basic Medical Sciences, Faculty of Medicine, Yarmouk University, Irbid, Jordan, yu.edu.jo; ^4^ Department of Biological Sciences, Al-Hussein Bin Talal University, Ma′an, Jordan, ahu.edu.jo; ^5^ Department of Microbiology, Graphic Era (Deemed to Be University), Dehradun, Uttarakhand, India, geu.ac.in; ^6^ Faculty of Medicine, Yarmouk University, Irbid, Jordan, yu.edu.jo; ^7^ Faculty of Medicine, University of Science and Technology, Irbid, Jordan, hust.edu.vn; ^8^ National University of Life and Environmental Sciences of Ukraine, Kyiv, Ukraine, nubip.edu.ua

**Keywords:** breast cancer, drug delivery, nanoparticles, redox-sensitive nanoparticles, stimuli-responsive nanoparticles

## Abstract

Breast cancer, one of the leading cancer types, directly contributes to cancer‐related mortality, but new therapies are required to improve treatment efficiency. Nanoparticle‐based strategies have already introduced the most radical advances in this regard, from their first formulation through their development to the next generation as promising candidates. The present review provides an overview of nanoparticle‐based strategies in breast cancer, their development, and their state of the art. The review addresses the diverse formulation of nanoparticles, including liposomes, dendrimers, and metallic nanoparticles, as well as their respective roles in drug delivery: bioavailability, focused therapeutic intervention, and lower systemic toxicity. This review covers studies on the engineering of nanoparticles for improved drug delivery, including cancer‐targeted delivery to tumors and optimization within the tumor microenvironment. Additionally, new nanosized drugs may also be utilized for novel modification of nanoparticle composition for combination therapeutics, which allows pharmacological agents to enter the tumor microenvironment by combined treatment with diverse types of other agents, to provide a synergistic, effective treatment in real time, in addition to real‐time monitoring. Stimuli‐responsive nanoparticles, which release the drug according to the appropriate stimulus signal to provide greater accuracy and regulation in delivering the drug, are also under investigation. This review notes trends in personalized nanomedicine and nanoparticle‐mediated immunotherapy that target personalized patient and immune responses, among others. Other exciting areas for nanoparticle research include AI‐based optimal design and sustainable biodegradable materials aimed at maintaining nanoparticle safety. Nanoparticle‐based therapies are a unique new frontier in breast cancer therapy. They have considerable clinical potential, offer promise for patient‐specific treatment, and warrant further investigation in breast cancer, particularly in advanced targeting mechanisms, multifunctional approaches, and individualized interventions.

## 1. Introduction

Breast cancer is one of the most common and challenging malignancies in females around the world, with approximately 2.3 million new cases annually diagnosed [1]. Despite advances in the early diagnosis and treatment of breast cancer, the heterogeneity and complexity of breast cancer biology remain a significant challenge for treatment. Classical therapeutic approaches (i.e., surgery, radiation, and chemotherapy) generally have limitations such as systemic toxicities, drug resistance, and inadequate targeting of tumor tissues [2]. The development of nanoparticles with a high surface area (1–100 nm) and easily modified surfaces that can overcome the wide variety of biological barriers has significantly advanced their therapeutic utility [3]. In this spirit, nanotechnology enhances drug delivery systems along with precision and controlled release [4]. Therefore, such high‐performance nanocarriers possess the ability to transport bioactive substances to the target cell [5]. Other advantages involve active targeting, increased circulation time, and decreased immune clearance through surface modification with biological ligands, biomimetic coatings, or zwitterionic or chitosan‐based materials [6].

Through local delivery (liposome or liposome‐type methods) and smart, sustained drug release systems, prolonged drug levels and minimized drug toxicity are achieved [7]. However, there still needs to be further work to realize how to transform these technologies to overcome issues such as patient heterogeneity, optimize nanoparticle design for precision medicine, and guarantee safety and scalability in clinical practice [8]. Ongoing research continues to refine these systems for more effective and personalized therapies across different diseases such as cancer and neurological disorders [3].

Nanoparticles can be prepared from different materials such as lipids, polymers, metals, and inorganic compounds [9]. This brings with it its own advantages and challenges with each type of nanoparticle. As an example, liposomal nanoparticles serve to make chemotherapeutic agents more bioavailable and stable, thus increasing therapeutic activity and decreasing potential adverse events [10]. With the advent of magnetic nanoparticles (MNPs) being used as powerful tools for cancer theranostics, their delivery can be guided by external magnetic fields directly to tumor sites. Moreover, their superparamagnetic properties enable external magnetic guidance for targeted drug delivery and enhance imaging performance, thereby offering dual therapeutic and diagnostic advantages. Moreover, MRI contrasts from MNPs help to observe the distribution of drugs and tumor response in real time [11]. Surface modifications of nanoparticles, including the addition of polymers, antibodies, or cell membranes, can amplify their interaction with cancer cells, increase their selective uptake, facilitate controlled or stimuli‐responsive drug delivery, and/or promote drug use [12]. Optimized therapy with these strategies overcomes physiological barriers, enhancing efficacy and reducing side effects in contrast with standard therapies [2]. For instance, chitosan‐ or folate‐coated MNP coatings have exhibited enhanced stability, targeted delivery, and controlled release of chemotherapeutics; biomimetic and ligand‐functionalized surfaces facilitate targeted tumor exposure and cell uptake [13].

This review evaluates the present state of nanoparticle‐based strategies for breast cancer pharmacotherapy. It will provide an overview of nanoparticle approaches and the methods that have been utilized to use these particles, as well as describe the progress made to date in preclinical testing and treatment. This review is not only a summary of recent advances but also a critical perspective on nanoparticle‐based strategies for breast cancer. We compare the relative strengths and weaknesses of major classes of nanoparticles and delivery concepts, evaluate the robustness and translational relevance of preclinical and clinical evidence, and highlight points of convergence or divergence among key studies [8, 14]. Special focus is placed on the approaches of the various platforms to overcome major clinical challenges such as systemic toxicity, multidrug resistance (MDR), and heterogeneity of the tumor microenvironment, with the goal to identify which approaches now appear most promising for clinical implementation [3, 5].

We apply an analytical framework that is specific to breast cancer and integrates the distinctive biological and clinical features of the disease. We start with a description of the potential for nanoparticle platforms to be tailored to the major molecular subtypes of breast cancer (hormone receptor–positive/HER2‐negative, HER2‐positive, and triple‐negative disease), focusing on subtype‐specific targets and resistance mechanisms [15]. Second, we discuss nanoparticle‐based strategies across the continuum of care, such as early stage neoadjuvant and adjuvant settings, locoregional control, and treatment of metastatic disease, emphasizing the role of disease stage and metastatic niches (bone, liver, lung, and brain) in driving design requirements for delivery systems [3, 14].

Third, we review the properties of stimuli‐responsive, combination, and immunomodulatory nanosystems for breast cancer–specific tumor microenvironmental features like dense stromal architecture, hypoxia, immune contexture, and tendency of MDR [5, 16]. In this context, each section below interprets advances in nanoparticle engineering and drug delivery with specific reference to their relevance and limitations in breast cancer, rather than oncology more generally [3, 17].

## 2. Nanoparticulate Systems in Cancer

**Figure 1:**
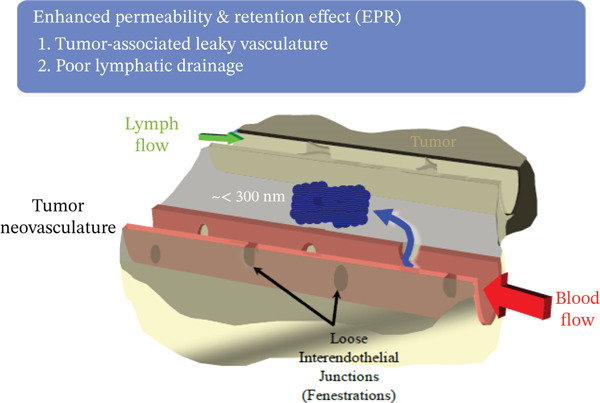
Passive tumor targeting facilitated by enhanced permeation and retention (EPR) effect. The EPR effect is a balance between enhanced tumor permeability and inadequate tumor interstitial fluid drainage that enables selective accumulation of the nanoparticles inside the tumor tissue.

Significant clinical applications of conventional chemotherapeutic agents are hindered, making their pharmacological action and efficacy limited [18]. In recent years, nanotechnology‐inspired strategies have shown promise as a solution to these shortcomings. For one thing, the synthesis of a sufficiently designed nanoparticulate system with the best particle size and half‐life can dramatically improve drug delivery [19]. These systems are able to reduce systemic chemotherapy side effects in treatment with the use of conventional chemotherapy; the systemic side effects may improve and improve patient efficacy through controlled drug accumulation and controlled release through a specific drug accumulation pathway [4]. As a result, targeted treatment is aimed at enhancing anticancer efficacy while minimizing damage to healthy tissues, thereby improving the overall safety and effectiveness of therapy [5]. Second, encapsulated drugs can be safeguarded by nanoparticles. By protecting these drugs from the biological environment, including metabolism of hormones and the interaction of serum proteins, the incorporation of nanoparticles maintains the drug stability and bioactivity of drugs during circulation [9]. This defense contributes to an improvement in the therapeutic activity of the drugs and the distribution of a higher concentration to the target sites [2]. Nanoparticles improve anticancer drug delivery partly through passive tumor accumulation [3]. When nanoparticles are engineered within an appropriate size range and circulation profile, they can preferentially accumulate in tumor tissue because of the leaky vasculature and poor lymphatic drainage that characterize many solid tumors [20]. This phenomenon, commonly described as the enhanced permeability and retention (EPR) effect (Figure 1), has historically been considered an important advantage of nanomedicine for breast cancer therapy [3, 20]. However, the magnitude of the EPR effect is highly heterogeneous across tumor types, disease stages, and patients, and passive accumulation alone is often insufficient to ensure therapeutically effective intratumoral drug concentrations [8, 20].

A second major advantage of nanoparticles is their potential to help overcome MDR, which remains a major cause of chemotherapy failure. MDR is commonly mediated by efflux transporters such as P‐glycoprotein that reduce intracellular drug accumulation and limit treatment efficacy [21, 22]. Nanoparticle‐based delivery systems can partially address this problem by facilitating intracellular uptake, protecting the payload during circulation, and enabling delivery pathways that reduce direct exposure of free drug to efflux‐mediated removal [5]. As a result, nanocarriers are being explored as promising tools for improving drug retention and restoring therapeutic response in resistant breast cancer cells [22].

A third advantage lies in the ability to engineer nanoparticle properties to improve targeting and controlled release rationally [12]. Parameters such as particle size, surface charge, ligand functionalization, and stimuli‐responsive coatings can be tuned to enhance tumor uptake, cellular internalization, and site‐specific drug release while reducing off‐target toxicity [3, 13]. In breast cancer, these design features support a broader strategy in which passive targeting, resistance modulation, and precision delivery are integrated to improve therapeutic efficacy more coherently than conventional formulations alone.

Nanoparticles are designed for targeted uptake, and their therapeutic effects reach their tissues effectively by selecting the correct size, surface, and drug release properties [3]. Studies in this area have shown that by tuning nanoparticle properties (e.g., particle size, surface charge, targeting ligands, and surface functionalization) and optimizing cellular internalization or tumor accumulation, improved cellular retention and therapeutic efficacy in the field of cancer, including breast cancer, have been reported [13]. As an example, drug uptake and controlled drug release in targeted nanoparticles to specific receptors (LDLR and dual EGFR/PD‐L1 targeting) or tumor microenvironment stimuli (pH and hypoxia) show enhanced cytotoxicity, tumor cell response, and reduced off‐target effects. Furthermore, smaller nanoparticles (e.g., 10–50 nm) accumulate more effectively in tumors and possess greater cellular uptake than their larger counterparts, providing additional reasons to use precise nanoparticle delivery methods for drug delivery efficiency [23].

As the recent findings highlighted, biomimetic coatings and stimuli‐responsive materials have emerged as one of the methods to optimize the target and release features that will eventually enable personalized and more effective therapies for cancer [12]. These results emphasize the contribution of nanoparticle engineering in progressing site‐specific, efficient, and safe therapeutic drugs that form part of modern drug delivery approaches [8]. For achieving optimal efficacy of the treatment, the optimal nanoparticulate drug delivery system can provide for the timing and localization of drug release. By doing so, this approach compensates for the drawbacks of MDR and enhances the precision behind drug delivery to minimize systemic toxicity and enhance therapeutic efficacy [22]. Taken together, the available literature indicates that nanoparticulate systems offer clear conceptual advantages over conventional formulations; however, the strength of evidence is uneven across different strategies [3]. EPR‐dependent passive targeting and size‐optimized carriers consistently improve intratumoral accumulation in preclinical models, yet their performance in heterogeneous human tumors is far more variable, and clinical benefit has been modest in some trials [8, 20]. Approaches aimed at overcoming MDR by bypassing efflux pumps are mechanistically compelling, but many studies rely on short‐term in vitro assays that may overestimate their impact in vivo and rarely benchmark against optimized standard‐of‐care regimens [5]. Similarly, while biomimetic coatings and surface‐functionalized nanocarriers can enhance circulation time and cellular uptake, they also introduce additional complexity for large‐scale manufacturing, reproducibility, and regulatory approval [6]. A critical appraisal, therefore, suggests that future research should prioritize rigorous in vivo validation, standardized endpoints, and head‐to‐head comparisons of nanoparticle designs under clinically relevant conditions. The advantage of nanoparticles in breast cancer drug delivery systems is given in Table 1.

**Table 1:** Advantages of nanoparticles in breast cancer drug delivery systems.

Advantage	Description	Applications in breast cancer
Enhanced drug bioavailability	Nanoparticles improve the solubility/stability of breast cancer drugs for better absorption [4]	Lipid‐based NPs enhance paclitaxel bioavailability [10]
Targeted drug delivery	Functionalized NPs target breast cancer cells, reducing off‐target effects [13]	HER2‐targeted NPs deliver doxorubicin to HER2+ cells [12]
Controlled release	NPs provide sustained release, prolonging action/reducing dosing [19]	Polymeric NPs for tamoxifen controlled release [24]
Reduced side effects	Targeted delivery minimizes healthy tissue exposure/systemic toxicity [2]	Gold NPs reduce toxicity at the tumor site [11]
Overcoming drug resistance	NPs bypass P‐gp efflux in resistant breast cancer [22]	Liposomes overcome MDR in breast cancer cells [21]
Improved imaging/diagnosis	NPs enhance imaging sensitivity/specificity for breast cancer [3]	Quantum dots/magnetic NPs for breast imaging [23]
Multifunctional capabilities	NPs combine delivery/imaging/therapy [9]	Dual‐function chemo + imaging NPs [20]
Personalized medicine	NPs tailored to subtypes/genetics [6]	Subtype‐specific targeted NPs [8]

Table 1 summarizes general advantages of nanoparticle‐based drug delivery, but not all advantages are equally validated or impactful in clinical settings in breast cancer, which has implications for decision‐making. In fact, improved bioavailability and decreased systemic toxicity are already confirmed by approved nanoformulations such as liposomal doxorubicin (DOX, Doxil) and albumin‐bound paclitaxel (PTX), which clinicians can choose favorably for patients at high risk of cardiotoxicity or hypersensitivity reactions [14, 25]. On the other hand, functions such as overcoming MDR or facilitating real‐time image‐guided therapy remain largely at the preclinical or early clinical level and should be viewed as promising but investigational alternatives that require careful patient selection and enrollment in clinical trials [3]. Therefore, when choosing a nanoenabled approach, clinicians and translational researchers should prefer platforms with demonstrated survival or quality‐of‐life benefits in Phase II–III trials and leave more complex multifunctional systems for hypothesis‐driven studies in which the incremental therapeutic gain justifies the additional cost and manufacturing complexity [3, 25].

## 3. Drug Release From Nanoparticles

Stimuli‐responsive nanocarriers (SRNs) are a new class of drug delivery systems developed to deliver pharmacological materials upon stimulation of specific environmental triggers [26]. Their surface‐level active characteristics can be responsive to a range of stimuli, such as endogenous (natural) or exogenous, leading to structural changes as well as selective drug delivery in regions of the body [27]. Endogenous signals are internal stimuli that the body releases when a physiological or pathological change has occurred in a part of the body. There may be a change in pH, reactive oxygen species (ROS), or enzyme concentration, which are usually classically correlated to the disease state.

Tumors often have a lower pH than healthy tissue surrounding areas, which can be explored by developing acid‐sensitive SRNs [17]. Likewise, the oxidative stress condition in tumors or inflamed tissues can activate the release of the drugs by ROS‐sensitive nanocarriers [26]. Increased concentrations of a few specific enzymes present in cancer, including matrix metalloproteinases, might also be used to induce drug release from enzyme‐sensitive nanocarriers. Using these endogenous stimulants, SRNs might offer targeted drug delivery that is designed for the disease microenvironment [17].

Exogenous stimuli, alternatively, external to the body, including heat, light, magnetic fields, or ultrasound (US), are additional external factors. These external stimuli facilitate the exact delivery of the drug for the pharmacologic system to the appropriate location or time, thus the time of drug release and the drug therapy [28]. In this regard, high‐light excitation, with near‐infrared radiation, can cause the drug release from photoresponsive nanocarriers, and US can promote the drug release from US‐sensitive carriers.

Stimuli‐responsive drug delivery systems would enhance their precision and effectiveness in delivering treatment [26]. Drug release or drug application through receptor binding, targeting the drug release into certain conditions in the body and external stimuli, would be helpful for minimizing the side effects and improving therapeutic results in clinical drug delivery [27].

### 3.1. Exogenous Stimuli

#### 3.1.1. Temperature‐Responsive Drug Delivery Systems (Thermoresponsive Nanocarriers)

Temperature fluctuations (both natural and induced) offer an important method of site‐specific delivery of chemotherapeutics. Thermoresponsive nanocarriers are engineered to take advantage of temperature changes, allowing targeted payload release. The abovementioned nanocarriers are made of polymers that can experience drastic changes in both their physical and chemical properties in response to changes in temperature, thus paving the way for controlled tissue delivery of the drug [29]. A popular thermoresponsive polymer is poly(*N*‐isopropylacrylamide) (PNIPAM), which was first described by Scarpa et al. in the 1960s. PNIPAM is found to undergo a phase transition around its lower critical solution temperature (LCST) [29]. Below this temperature, PNIPAM is hydrophilic and stays hydrated. When the temperature becomes above the LCST, PNIPAM is then hydrophobic, leading to polymer collapse and encapsulated drug liberation. This phase change can be initiated by endogenous changes in the temperature in diseased tissue or by an externally applied heat. Poly(*N*‐vinylcaprolactam) (PNVCL) is still a well‐studied thermoresponsive polymer and has been known for its biocompatibility due to profound conformational changes to temperature [10]. After grafting to chitosan, these polymers are assembled into hybrid nanoparticles (e.g., chitosan‐*g*‐PNVCL) that allow temperature‐dependent drug release at the site, showing improved cancer cell toxicity to curcumin‐based systems past their LCST range [30].

For thermoresponsive nanocarriers, the temperature range used for responsiveness must be tuned and optimized, generally between 37°C and 42°C, as this range ensures a good drug release and less toxicity due to the excessive temperature that can denature the protein [29]. Researchers should adjust the temperature response of these nanocarriers to satisfy their therapeutic needs by modulating the hydrophilic/hydrophobic property of the polymer.

#### 3.1.2. Light‐Responsive Nanocarriers

Light‐responsive nanocarriers provide one of the advanced methods for controlled drug release in drug delivery systems. In these systems, light‐sensitive systems are utilized to modulate the release of the drug by cleaving the linker upon light exposure or modifying carrier properties using molecular switches, such as azobenzene [31]. Peng et al. worked on a light‐responsive hydrogel system of dextran attached with azobenzene groups and mixed with cyclodextrin‐decorated dextran. In the case of azobenzene, the trans configuration of it fits into cyclodextrin cavities with cross‐linking, allowing for gelation. During the light irradiation, azobenzene isomerizes to the cis configuration, interferes with fitting into cyclodextrin cavities, and results in cross‐link dissociation and drug release [32]. The light‐responsive hydrogels described in this system are suitable for controlled drug delivery. One common approach is to embed a light‐sensitive linker into the nanocarrier. After exposure to a given wavelength of light, this linker is broken, and so the encapsulated drug is released.

One important limitation of light‐responsive nanocarriers is the possibility of unintended premature drug release before deliberate irradiation, which is more accurately described as dark leakage or insufficient off‐state stability rather than true light activation. Such premature release may result from limited nanocarrier stability under physiological conditions, nonspecific cleavage of photosensitive linkers, or background reactive species, ultimately causing off‐target toxicity and reducing therapeutic precision. Therefore, an ideal light‐responsive nanocarrier should remain stable during circulation and release its payload only after spatiotemporally controlled light exposure at the tumor site.

As an example of a successful light‐triggered system, Yuan et al. developed light‐responsive AIE nanoparticles to overcome DOX resistance in breast cancer cells. In that system, irradiation generated ROS that cleaved the thioketal linker and promoted cytosolic drug release, thereby enhancing therapeutic efficacy in resistant MDA‐MB‐231 cells. This study illustrates the therapeutic potential of photoactivated nanocarriers, but it should not be cited as evidence of premature drug activation or dark toxicity.

Another example describes a novel light‐dependent micellar drug delivery system by conjugated coumarin block copolymers. The polymers poly(ethylene oxide)‐*b*‐poly(*n*‐butyl methacrylate‐*co*‐4‐methyl‐[7‐(methacryloyl)oxyethyloxy]coumarin) (PEO‐*b*‐P(BMA‐*co*‐CMA)) were prepared through atom transfer radical polymerization. Drugs in the micelle were loaded with the anticancer drug 5‐fluorouracil (5‐FU), which was covalently bound to coumarin by UV irradiation (> 310 nm). UV irradiation (254 nm) was found to control the release of 5‐FU in vitro studies, proposing that such systems could successfully deliver anticancer agents with low toxicity in normal cells [28].

#### 3.1.3. US‐Responsive Nanocarriers

US has long been used in medical imaging for its noninvasive penetration of tissues and its precision in targeting well‐defined points [33]. For drug delivery, US frequencies exceed the audible range (> 20 kHz), with high‐intensity focused ultrasound (HIFU) operating between 0.8 and 3.5 MHz being particularly useful for penetrating tumors without significant tissue injury [34].

Polymeric materials that respond to US stimuli are one of the important challenges for the development of US‐responsive nanocarriers. Polymeric micelles, including Pluronic P105 and PEG–polyester block copolymers, have been investigated and shown that US can cause drug release via physical processes such as cavitation, leading to transient micelle disruption and payload diffusion rather than polymer degradation [33]. This mechanism of release represents a controlled release mechanism that can be activated by US, highlighting its promising application in noninvasive drug delivery systems [34].

Subsequent studies by Zhang et al. investigated the behavior of hydrophobic Nile Red dye encapsulated in degradable and biocompatible PLA‐*b*‐PEG block copolymer nanoparticles. Their studies showed that the release of the dye was dependent on both the duration and intensity of HIFU exposure, with irreversible polymer degradation observed [35]. The decrease in molecular weight of the PLA‐*b*‐PEG polymer during HIFU treatment confirmed that these nanoparticles could be utilized in systems where polymer degradation is induced by US, providing a viable approach for targeted drug delivery [35].

#### 3.1.4. Magnetic‐Responsive Nanocarriers

The emergence of magnetic‐responsive nanocarriers in recent years has raised attention for their promise for enhancing therapeutic efficacy and drug delivery in clinical and biomedical settings (Parihar et al.[36]). These nanocarriers exploit the extraordinary properties of magnetic fields (remote control, deep tissue penetration, etc.), which are particularly beneficial to targeted therapeutics (Parihar et al.[36]). Several iron oxide–based MNPs have been investigated for clinical applications and shown good safety characteristics as well as great potential to be used in imaging, targeting, and treatment of a wide variety of diseases, including cancer. Superparamagnetic iron oxide nanoparticles (SPIONs) are the most investigated forms of magnetic nanocarriers. These nanoparticles are broadly utilized for drug‐selective targeting and magnetically mediated hyperthermia, in which oscillating magnetic fields (OMFs) can produce localized heating to accelerate the transportation of a drug or ablate cancer cells [37]. However, recent studies utilized SPIONs with lower magnetic field strengths that enhanced the drug delivery even across biological barriers to inhibit unwanted heating of human tissues and facilitate repeat drug delivery for chronic diseases by allowing multiple treatments [38]. The optimization of the structure of nanoparticles (shape anisotropy, surface functionalization, and other advanced nanoparticle construction concepts) has increased the biocompatibility, targeting accuracy, and drug release control properties (Parihar et al.[36]).

Magnetic nanocarriers facilitate the diffusion of medicine and enable drug penetration through obstacles such as mucus or biofilm (common in cystic fibrosis and some infections). An example of applications like MNPs in cystic fibrosis is to disturb thick mucus and obtain drugs through their nanoparticles, so they can be used to provide an adequate therapeutic advantage. Previous studies have demonstrated the ability of these nanoparticles to be targeted at target sites using external magnetic fields, promoting mechanical or thermal disruption of biofilms and mucus barriers, further enabling their delivery to organs [38]. Notably, iron oxide–based MNPs have been shown to be capable of magnetically directed, imaging‐tracked, and trigger‐controlled drug release, enhancing penetration and therapeutic activity even in harsh environments [37]. In addition, the recent advancements in materials engineering have made it possible to develop multifunctional magnetic nanocarriers with the combined targeting aspect, imaging application, and responsive drug release to make them especially effective in addressing biological barriers (Parihar et al.[36]).

In addition, MNPs are actively studied by researchers to deliver controlled drug release. For example, drug‐conjugated MNPs release their payloads after exposure to a magnetic field, which offers spatial control of drug delivery and temporal control [37]. This enables fine‐tuning of the targeting with less off‐target impact, and it would be an attractive strategy for increasing the treatment effect of medication (Parihar et al.[36]).

### 3.2. Endogenous Stimuli

#### 3.2.1. pH‐Responsive Drug Release

pH‐responsive nanocarriers are engineered to adapt to changes in pH normally described in pathological conditions, for example, in the acidic microenvironment of tumor bodies or their intracellular compartment. By using the impact of pH on nanocarrier motion, this method facilitates the selection of therapeutic drugs and drug excretion with the benefit of increased therapeutic activity against systemic side effects [39]. Tumor microenvironments are generally more acidic than normal tissues due to extremely high glycolytic activity and lactic acid accumulation. In this acidic environment of pH 6.2–6.9, it makes it possible to formulate nanocarriers that deliver their therapeutic payload to tumor tissues [40].

Intracellular compartments like endosomes and lysosomes have acidic pH (4.0–6.0), but these may be utilized for inducing drug mobilization from pH‐responsive nanocarriers [39]. pH‐responsive nanocarriers are polymers whose solubility will vary in response to the pH shift mechanism. Polymers with amine or carboxylic acid groups have the potential to convert from hydrophobic to hydrophilic polymer, depending on the reaction, in protonation and deprotonation. This transition can also cause the breaking of the micellar structure and release of the drug in acid. Chitosan, another well‐known polymer for pH‐responsive systems, has been utilized to develop novel nanocarriers for releasing DOX in a pH‐responsive system [24]. For instance, Matalqah et al. developed *O*‐carboxymethyl chitosan phthalate (OCMCS‐Ph) nanoparticles that produced DOX efficiently in the acidic tumor microenvironment, which exerted a superior therapeutic effect and minimized the systemic adverse reaction [40].

pH‐responsive nanocarriers are manufactured using a layer‐by‐layer (LbL) assembly method consisting of multiple active layers. These nanocarriers typically consist of a core into which the drug is loaded, a polyelectrolyte multilayer which is designed to respond to the pH changes, and a stealth layer added to prolong circulating time [24]. The pH sensitivity is a consequence of protonation and deprotonation of charged groups in the multilayer. For example, Chai et al. [24] have used chitosan and alginate in LbL assembly to induce DOX release from poly(lactic‐*co*‐glycolic acid) (PLGA) nanoparticles, as well as the capability of drug release.

#### 3.2.2. Redox‐Responsive Nanoparticles

Redox‐responsive nanocarriers are complex delivery systems that are sensitive to redox conditions within cells where the drugs normally occur. These nanocarriers use these redox conditions (e.g., varying, reducing, and oxidizing agent concentrations) to induce drug release and specifically can allow for the release of drugs that target the cytosol or nucleus of their target cells [41]. The specific characteristics of these nanocarriers are particularly useful for achieving specific therapeutic effects and reducing off‐target side effects [41]. The intracellular environment has a greater concentration of reducing agents than the extracellular environment. Glutathione (GSH), one of the dominant biological reducing agents, is found at the cellular level at significantly greater concentrations than the extracellular environment (approximately 2–10 mM compared with the extracellular concentration of ~2–20 *μ*M), respectively [41]. This gradient in concentration is leveraged to engineer redox‐responsive nanocarriers, which remain stable in the extracellular milieu yet undergo perturbation upon the expression of high levels of GSH in the intracellular environment [41].

Redox‐responsive nanocarriers can exploit disulfide (–SS–) and diselenide (–SeSe–) bonds, which are stable under normal extracellular circumstances but are reduced to thiols (–SH) and selenols (–SeH) by intracellular reducing conditions [41]. Disassembly of the nanocarrier and release of the therapeutic payload result from these reductions. Some key examples of such redox‐responsive nanocarriers are as follows.

##### 3.2.2.1. Polymer‐Based Nanocarriers

Yang et al. [41] developed a redox‐sensitive amphiphilic polymer, heparin‐alpha‐tocopherol succinate (Hep‐cys‐TOS), which was synthesized by grafting hydrophobic TOS to heparin using a redox‐sensitive linker, cystamine. This polymer self‐assembled into nanoparticles with a core–shell structure. In the presence of high GSH concentrations in tumor cells, the nanoparticles disassemble, leading to burst release of PTX and subsequent induction of cell apoptosis. These nanoparticles demonstrated desirable properties, including suitable particle size, low cytotoxicity, and enhanced anticancer activity in vitro [42].

##### 3.2.2.2. Nucleic Acid Delivery

Nucleic acid delivery systems (e.g., redox sensitive, amido, and histidine polycations) are starting to emerge in gene therapy [43, 44]. A notable discovery is the use of cationic polymers: poly(disulfide amine), disulfide‐containing poly(amido amine), and histidine polycations. These polymers then form stable complexes with either the negatively charged DNA or RNA, which can be released by the redox conditions inside the cell [17, 45].

#### 3.2.3. Oxidation‐Responsive Drug Release

The intricate balance of ROS within biological systems is crucial for maintaining cellular function [42, 46]. However, when this state is disturbed, as is the case, elevated levels of ROS can give rise to oxidative stress, which is a pathological condition characteristic of cancer and inflammation and is often found in various pathological conditions [47]. In that way, the ROS presents a unique opportunity both for research and for practice: Nanocarriers that can respond specifically to these types of oxidative environments can now be designed and utilized [48]. By providing nanocarriers to directly attack harmful ROS‐polluted microangiogenesis pathways with their oxidative response, a unique opportunity exists [27]. Oxidation‐mediated drug delivery systems utilizing oxidative ROS may provide a new class of ROS drug delivery devices to deliver to patients a controlled and effective drug release [17]. These advanced nanocarriers are designed into molecular forms that can change chemical structures and chemically respond to oxidative stress, such that they can give off a precise release of drugs at the site of disease [49]. This strategy employs the nature of individual chemical linkages or functional groups in ROS of different classes present within a nanocarrier to the functional groups for enhanced drug release to ROS in line with a methodical and efficient improvement in drug transport and efficacy when drug delivery is required during difficult clinical trial situations [50]. ROS consist of molecules such as hydrogen peroxide (H_2_O_2_), superoxide anions (O_2_^-^), hydroxyl radicals (‐OH), and singlet oxygen (^1^O_2_) [47]. Elevated levels of ROS are frequently correlated with disease conditions, such as cancer, characterized by oxidative stress [45].

Oxidation‐responsive nanocarriers respond to the oxidative environment by a chemical mechanism when their payload is released [27]. These nanocarriers have specially selected chemical bonds or functional groups to be oxidative sensitive [47]. Depending on the mechanism of oxidation‐responsive release, there are three different types of nanoparticles.

##### 3.2.3.1. Disulfide Bond–Based Nanocarriers

In oxidation‐responsive systems, disulfide bonds (–S–S–) dominate [51]. ROS cleaves these bonds, releasing the drug [49]. Such as when ROS are present, disulfide bonds are converted to thiol groups (–SH), destroying nanocarriers and the cell [50]. A class of nanoparticles evolved based on disulfide bonds for an oxidation‐sensitive process. The objective of these nanoparticles was to release their payload in response to high ROS levels [48]. The nanoparticles were found to deliver drugs in oxidative media, as exhibited by cancerous tissues.

##### 3.2.3.2. H_2_O_2_‐Sensitive Nanocarriers

Some nanocarriers use linkers sensitive to H_2_O_2_ [46]. Under the oxidative conditions of the system, these linkers can be degraded and released by the encapsulated drug developed H_2_O_2_‐responsive nanocarriers [17]. These nanocarriers contained peroxide‐sensitive linkers that degrade under the influence of H_2_O_2_ to have the encapsulated drugs released in a controlled manner [27]. These nanocarriers exhibited application potential in oxidative stress–related diseases.

##### 3.2.3.3. Oxidation‐Sensitive Polymers

This is because oxidation can induce changes in cross‐linking of polymer networks in the nanocarrier [50]. For instance, the disruption of cross‐links leading to oxidative cleavage of the cross‐links within a polymeric matrix could remove the nanocarrier and release the therapeutic agent, investigating reactive groups of oxidation‐sensitive polymers that are reactive to ROS [49]. These polymers might undergo structural alterations upon oxidation and reactively liberate therapeutic drugs [48]. This strategy was specifically well‐suited for the treatment of diseases caused by oxidative stress, inflammation, and cancer [45].

The potentials of oxidative‐responsive nanocarriers in oxidative stress–related diseases. Oxidation‐responsive nanocarriers may pave the way for targeted therapy [47]. Their mechanism of action in a well‐defined oxidative stress environment in cancer, inflammation, and other diseases is unique for the release of drugs [17]. Further studies will address the optimization of these systems for clinical use, to further solidify these systems and to develop broad applicability for diseases [49].

Redox‐responsive polymers are currently also being investigated for the delivery of nucleic acids [44]. Various redox‐sensitive cationic polymers like poly(disulfide amine), disulfide‐containing poly(amido amine), and histidine polycations have shown promise in gene therapy [43]. These polymers can facilitate the delivery of nucleic acids by forming stable complexes with the negatively charged DNA or RNA, which are then released in response to the redox conditions inside the cell.

### 3.3. Clinical Translation, Toxicity, and Real‐World Feasibility of SRNs

Many types of nanocarriers responsive to exogenous and endogenous stimuli have shown improved drug release control, tumor accumulation, or reversal of MDR in vitro and in animal models. However, their successful translation into routine clinical breast cancer care remains limited [52]. Most of the nanomedicines that have progressed to late‐stage clinical trials or regulatory approval have been based on relatively simple liposomal or albumin‐bound formulations, with more complex “smart” systems with multiple responsive components still largely confined to preclinical evaluation [53]. This translational gap is indicative of the biological complexity of human tumors and the significant challenges to reproducibly generate sufficient stimulus magnitude (e.g., heterogeneous pH gradients, variable redox status, and limited penetration of light and US) across all lesions in an individual patient [54].

Some specific platforms have been created to promote progress. Examples include lyso‐thermosensitive liposomal DOX (ThermoDox) combined with local hyperthermia that has moved into early phase clinical trials [55]. Intravascular drug release at 40°C–42°C in preclinical models led to several‐fold higher intratumoral DOX concentrations than conventional formulations and is currently being tested in breast cancer patients with MR‐guided HIFU [55, 56]. Similarly, thermosensitive liposomes encapsulating platinum drugs demonstrated approximately a twofold increase in cytotoxicity under mild hyperthermia (40°C) relative to normothermia (37°C) in MDA‐MB‐231 cells, exemplifying that “enhanced efficacy” is often associated with discrete, quantifiable improvements in specific experimental contexts rather than general effects across all settings [56]. However, in clinical practice, these advantages must be balanced with the practical aspects of specialized hyperthermia equipment, accurate temperature measurement, and careful patient selection to prevent off‐target heating in real‐world oncology centers.

Other critical determinants of clinical feasibility are safety, toxicity, and manufacturability of SRNs [53]. Complex architectures comprising polymers, inorganic cores, targeting ligands, and stimulus‐sensitive linkers pose a risk of unexpected biodistribution, long‐term accumulation in tissues, and immunotoxicity, particularly for cationic polymers and some inorganic materials [57]. This requires detailed nanotoxicology assessment from regulatory bodies, including standardized in vitro, ex vivo, and in vivo assays, as well as robust control of batch‐to‐batch reproducibility, impurity profiles, and scalability of manufacturing processes. Meeting these requirements can be more challenging for multicomponent, stimuli‐responsive systems than for simpler nanoparticles, which partly explains why many “advanced” constructs remain at the proof‐of‐concept stage despite promising laboratory results [53].

Accordingly, descriptors such as “promising,” “advanced,” or “effective” for stimuli‐responsive nanoparticles should be understood in the context of well‐defined benchmarks, such as fold changes in intratumoral drug concentration, reduction in systemic exposure, or improvement in tumor growth inhibition or survival in animal models, rather than as indications of established clinical benefit [57]. Currently, most of the evidence for SRNs in breast cancer is preclinical or early phase, and their therapeutic advantage over approved nanomedicines remains to be proven in adequately powered randomized trials that capture not only efficacy but also toxicity, cost‐effectiveness, and integration with existing standards of care [52, 58]. A more critical and quantitatively anchored appraisal of these systems might help align future research with clinically meaningful endpoints and allow rational prioritization of candidates for translation [57].

## 4. Types of Nanoparticles

Nanoparticles are minute particles that have attracted great attention in several domains thanks to their distinctive physical and chemical properties [3]. These properties are often quite dissimilar from bulk material properties, driving a new development space in medicine, electronics, environmental studies, materials engineering, and others [14]. In addition to classifying nanoparticle platforms by material and function, it is equally important to distinguish between systems that are already used clinically and those that remain investigational.

### 4.1. Clinically Approved Nanoparticle Formulations and Their Practical Advantages

A clinically grounded perspective is important, as few of the nanoparticle formulations have made the leap from promising laboratory systems to products approved for patient use or under clinical evaluation [3, 57]. The most established examples in breast cancer and related solid tumor practice include pegylated liposomal DOX (Doxil/Caelyx), nonpegylated liposomal DOX (Myocet), albumin‐bound PTX (Abraxane), and polymeric micelle PTX formulations such as Genexol‐PM and Nanoxel M in some markets [59, 60].

The main practical advantage of nanomedicine is exemplified by these clinically used nanoformulations, which often improve drug solubility, alter pharmacokinetics, reduce exposure to toxic excipients, and in some settings improve tolerability relative to conventional formulations [3]. For example, Abraxane was developed as a solvent‐free albumin nanoparticle formulation of PTX to avoid Cremophor EL–associated toxicity. In contrast, liposomal DOX formulations help modify tissue distribution and can reduce some aspects of systemic toxicity, especially cardiotoxicity risk relative to free DOX [59].

These approved products also reflect, at the same time, the current limitations of the field. Indeed, the clinical benefit has been more evident in safety, convenience of formulation, or pharmacokinetic improvement rather than in dramatic gains in overall survival. Issues such as hand‐foot syndrome with pegylated liposomal DOX, variable tumor accumulation, cost, and complexity of manufacture remain important barriers to wider impact [61].

In addition to approved systems, several nanoparticle platforms are still under clinical development, such as polymeric nanoparticles, thermosensitive liposomes, albumin‐based derivatives, and some inorganic platforms [3]. Examples discussed in the literature are BIND‐014, a docetaxel‐loaded polymeric nanoparticle that has been evaluated in early clinical studies, and ThermoDox, a heat‐triggered liposomal DOX platform that is being tested with local hyperthermia approaches. In contrast, newer inorganic approaches, such as gold nanoparticles, remain far less mature clinically [62].

Overall, the current clinical landscape suggests that the most translatable nanoparticle technologies for breast cancer are still simpler and better characterized platforms, especially liposomal and albumin‐bound systems [3]. More complex stimuli‐responsive, targeted, or multifunctional nanocarriers are scientifically attractive, but still need stronger evidence for reproducible tumor delivery, scalable manufacturing, long‐term safety, and clear superiority over existing standards of care before they can be considered routine clinical options.

### 4.2. Translational Challenges, Regulatory Considerations, and Clinical Limitations

Table 2. Types of nanoparticles, description, exemplars, use cases, advantages, and limitations. From translational standpoints, major nanoparticle platforms discussed in this review show strikingly different levels of clinical validation, reasons for failure of trials, and regulatory or manufacturing hurdles [3, 61]. Liposomal and albumin‐bound formulations such as pegylated liposomal DOX (Doxil/Caelyx) and albumin‐bound PTX (Abraxane) are the most clinically advanced platforms in breast and other solid tumors, reflecting relatively mature manufacturing processes and well‐characterized safety profiles [59]. In contrast, polymeric micelles and inorganic or metallic nanoparticles have been included in a number of early phase cancer trials; however, the advancement beyond Phase II is limited, which contributes to the high attrition rate seen for cancer nanomedicines overall [61]. Reasons for the failure of trials across platforms include modest improvements in overall survival compared with standard regimens, unexpected toxicities, suboptimal tumor accumulation, and difficulty in demonstrating clear clinical benefit despite improved pharmacokinetics [57].

**Table 2:** Prominent types of nanoparticles for breast cancer therapy.

Type of nanoparticle	Description	Examples	Applications	Advantages	Disadvantages
Liposomes	Spherical vesicles are composed of lipid bilayers [10]	Doxil (liposomal doxorubicin)	Delivery of chemotherapeutic agents; reduced systemic toxicity [4]	Biocompatible/biodegradable; encapsulate hydrophilic/hydrophobic drugs [9]	Limited loading; premature release [3]
Polymeric nanoparticles	Biodegradable polymers like PLGA, PCL, and PLA [19]	PLGA NPs with paclitaxel [24]	Drug/gene/protein carriers; controlled release [8]	Versatile; biocompatible release control [2]	Synthesis complexity; degradation issues [18]
Gold nanoparticles	Metal NPs with various shapes/sizes [11]	AuroShell (gold nanoshells)	Imaging; photothermal/targeted delivery [13]	Photothermal properties: Customizable [12]	Toxicity, cost, and interactions [2]
Quantum dots	Semiconductor NPs with optical properties [23]	Qdot (Invitrogen)	Imaging/diagnostics with ligands [3]	Sensitive imaging; photostability	Toxicity; biocompatibility; costs
Magnetic nanoparticles	Superparamagnetic iron oxide (SPIONs) [11]	Ferumoxytol (Feraheme) [37]	MRI; targeted delivery; hyperthermia (Parihar et al.[36])	Imaging/therapy; magnetic guidance [38]	Degradation; toxicity
Dendrimers	Branched nanoscale polymers [4]	PAMAM dendrimers	Drug/gene delivery; imaging [13]	Size control; high loading [9]	Cost; toxicity; synthesis
Silica nanoparticles	Mesoporous silica (MSNs) with pores	MSN@DOX	Controlled release; targeted imaging [17]	High surface/stability; tunable release [39]	Silica toxicity: Production scale
Hybrid nanoparticles	Organic/inorganic combinations [6]	Silica‐coated gold with drugs	Multimodal imaging/therapy/delivery [12]	Enhanced properties: Versatile [3]	Design complexity: Responses
Protein‐based nanoparticles	Self‐assembled from albumin/casein	Abraxane (albumin‐paclitaxel)	Drug delivery: Stability/release [41]	Biocompatible; reduced effects [4]	Size control; instability

These systems are also more complex to regulate and manufacture, as the multicomponent constructs (liposomes, polymeric particles, and hybrid nanoarchitectures) require tight control of critical quality attributes such as particle size, surface charge, drug loading, and release kinetics to ensure batch‐to‐batch reproducibility during scale‐up [3, 9]. Regulatory agencies have explicitly addressed these concerns in guidance for liposomal and other nanoenabled drug products, emphasizing the need for robust process analytical technologies and detailed physicochemical characterization to mitigate variability and immunogenicity risks [63]. Collectively, these observations suggest that whereas several platforms are promising, clinically validated success is restricted to a few technologically and regulatory mature formats, highlighting the need to consider clinical translation, regulatory acceptability, and scalable manufacture in the design of next‐generation breast cancer nanomedicines from the outset [3].

### 4.3. Comparative Evaluation and Future Translational Perspectives of Nanoplatforms

Looking across the various nanoparticle platforms, a number of priorities and tradeoffs come to the fore. Liposomes and polymeric nanoparticles have the most mature clinical experience and tend to have favorable biocompatibility and scalable manufacturing, making them strong near‐term candidates for breast cancer applications. Metallic and quantum dot systems have better imaging and photothermal properties, but are more concerning regarding long‐term toxicity, biodistribution, and clearance, which may limit their use to carefully selected indications or combination regimens [57]. Dendrimers, mesoporous silica, and hybrid organic–inorganic constructs allow sophisticated multifunctional designs, but their structural complexity may hinder quality control and regulatory approval [3]. From a translational perspective, the most functionally sophisticated platforms, with established safety, manufacturability, and regulatory pathways, are most likely to progress rapidly toward routine clinical implementation in breast cancer. We hope to shed light on the many ways in which nanoparticles facilitate technological development and solve global problems in the face of the risks and barriers involved. Looking at these factors [9].

**Figure 2:**
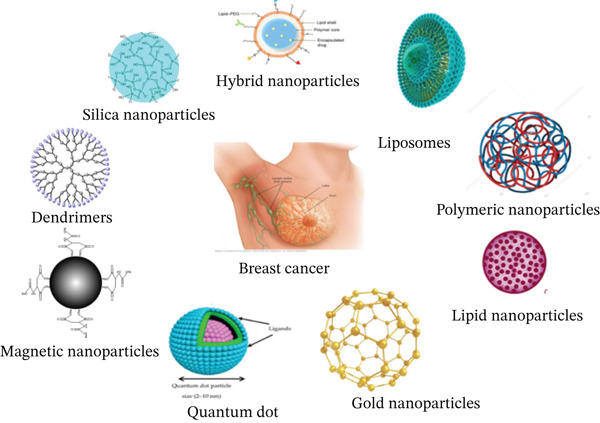
Types of nanoparticles used in breast cancer treatment.

Here are some of the most common nanoparticles used for drug delivery to breast cancer (Figure 2) [14]. From a translational point of view, Table 2 also serves as a practical decision‐making framework to select a suitable nanoplatform for a given breast cancer indication. Liposomes and protein‐based nanoparticles (e.g., Abraxane) are already in clinical use and are appropriate as first‐line nanocarriers when the goal is to improve pharmacokinetics or reduce toxicity of conventional chemotherapeutics without majorly modifying the treatment paradigm [59, 64]. Polymeric nanoparticles and mesoporous silica systems provide better control over drug release and multiplex loading, but most are in a preclinical or early clinical development stage.

They are more suitable for combination therapy designs or precision delivery concepts tested in translational studies [3]. Metallic and quantum dot platforms offer powerful imaging and photothermal capabilities. However, their long‐term toxicity and clearance profiles are less well defined, suggesting that they should be prioritized for short‐term diagnostic or theranostic applications, rather than chronic systemic administration at present [57]. Hybrid and biomimetic nanoparticles are promising candidates to overcome biological barriers and immune clearance, but still face significant manufacturing, scalability, and regulatory challenges and therefore should be considered next‐generation candidates that require rigorous comparative studies against simpler, clinically established systems [3].

## 5. Recent Approaches and Future Perspectives in Nanotechnology

Future work on nanoparticle‐based therapies for breast cancer is likely to build on the most clinically advanced platforms—such as liposomal, polymeric, and protein‐based nanocarriers—by refining targeting strategies, optimizing pharmacokinetics, and integrating robust safety evaluation, rather than relying on entirely new and speculative concepts [57, 65]. In the near to midterm, realistic priorities include improving subtype‐specific delivery (e.g., HER2‐positive and triple‐negative disease), designing trials that directly compare nanoparticle formulations with current standards of care, and establishing standardized characterization and reporting frameworks that facilitate regulatory review and cross‐study comparison [19]. Against this background, emerging themes such as personalized nanomedicine, biorthogonal chemistry, and artificial intelligence (AI) are discussed in this section as enabling tools that can support rational nanoparticle design and patient stratification, provided they are grounded in experimentally validated mechanisms and evaluated through rigorous preclinical and clinical studies [3, 66].

### 5.1. Personalized Nanomedicine

Most likely, personalized nanomedicine in breast cancer will progress by correlating nanoparticle design with well‐defined molecular subtypes and validated biomarkers rather than by following broadly framed, highly individualized ideas [67]. In practice, this means designing carriers to take advantage of well‐understood markers such as overexpression of HER2, hormone receptor status, BRCA mutation, or PD‐L1 expression, to improve drug accumulation in specific patient populations relative to conventional formulations [68]. Current preclinical research shows that ligand or antibody‐functionalized nanoparticles may enhance the delivery of chemotherapeutics or biologics to HER2‐positive or triple‐negative models, giving a solid foundation for future subtype‐focused clinical trials [69].

To translate personalized nanoparticle strategies into the clinic, important steps forward include (i) prospectively adopting companion diagnostics or liquid biopsy readouts to identify patients most likely to benefit, (ii) designing trials comparing nanoparticle enabled regimens versus biomarker matched standard therapies, and (iii) integrating pharmacokinetic, pharmacodynamic, and toxicity data to optimize dosing in specific subpopulations [68, 70]. In this context, data‐driven approaches, such as AI, should be considered complementary tools for the analysis of large multiomic and imaging datasets to identify targets and stratify patients, with predictions validated in robust experimental models and eventually in well‐controlled clinical studies. Table 3. Summary of recent approaches in personalized nanoparticle design [71].

**Table 3:** Emerging strategies in personalized nanoparticle design for breast cancer.

Strategy	Description	Examples of breast cancer
Stimuli responsive	pH/ROS/redox‐triggered release [17]	Acid‐sensitive DOX release [40]
Surface functionalization	Ligands (folate, antibodies) for targeting [12]	HER2/chitosan‐coated MNPs [13]
Biomimetic coatings	Cell membrane mimics evade immunity [6]	Prolonged circulation [3]
AI‐optimized design	Personalized via patient data [8]	Subtype‐specific formulations [1]

Table 3 not only summarizes emerging design strategies but also highlights which concepts are closest to clinical translation and where the major failure risks are. While stimuli‐responsive and surface‐functionalized systems have reached early phase trials, their success will require robust validation of tumor‐specific triggers and biomarkers in heterogeneous breast cancer populations [72]. Biomimetic coatings and AI‐optimized formulations remain largely preclinical proof of concept. These may provide the most personalization but require sophisticated manufacturing, regulatory acceptance of complex release mechanisms, and prospective demonstration that they outperform simpler nanoformulations in defined patient subgroups [73]. So for immediate clinical translation, researchers could concentrate on stimulus‐responsive or ligand‐targeted platforms with obvious companion diagnostics and manageable quality control workflows, reserving complex AI‐guided and biomimetic systems for exploratory or basket trials where iterative design might be driven by adaptive learning [57].

### 5.2. Combination Therapy Nanoparticles

Developing nanoparticles for the delivery of multiple therapeutic agents or therapy with imaging agents represents a remarkable development in the field of cancer drug treatment [74]. The use of multifunctional nanoparticles also permits the simultaneous transfer of chemotherapeutics, targeted therapies, and imaging agents. The combination of the two allows synergistic effects to achieve better therapeutic results and better monitoring of therapy response. As an example, nanoparticles that contain DOX and trastuzumab, as well as functional imaging capabilities, can be used to monitor and adjust therapy in real time.

### 5.3. Stimuli‐Responsive Nanoparticles

Innovations in nanoparticles that are activated by a whole range of internal stimuli (i.e., certain enzymes or temperatures beyond the classical pH and redox reactions) will allow for an efficient means of drug delivery. By exploiting distinct biological states within tumors or their microenvironment, these nanoparticles can allow controlled drug release. This specificity alleviates off‐target effects and enhances the drug′s therapeutic effect. For example, enzyme‐sensitive nanoparticles release drugs when specific overexpressed enzymes are present in breast cancer cells [16].

### 5.4. Nanoparticle‐Mediated Immunotherapy

Nanoparticle construction to improve the delivery and treatment of immunotherapeutics has led to innovative strategies for anticancer treatment [16]. Nanoparticles can be crafted to transport immune checkpoint inhibitors or cancer vaccines into tumor or antigen‐presenting cells. This focused delivery can induce greater immune responses directed toward tumors and may thus improve treatment efficacy in immunotherapy. Nanoparticles loaded with PD‐1/PD‐L1 inhibitors or containing tumor antigens could generate a strong immune response against tumor antigens when they are administered.

### 5.5. Targeted Delivery to Metastatic Sites

These complex drug delivery sites must be targeted by the identification and the use of either unique markers or environmental conditions at metastatic locations, thereby making therapy for metastatic breast cancer much more effective and minimizing systemic side effects. For instance, nanoparticles can target specific adhesion molecules or biomarkers mapped on specific metastatic lesions [75].

### 5.6. Biorthogonal Chemistry for Nanoparticle Functionality

Biorthogonal chemistry offers a set of highly selective reactions that can, in principle, be used to fine‐tune nanoparticle properties or to trigger drug activation in vivo, but most applications currently remain at an early preclinical stage [76]. Rather than treating biorthogonal strategies as a generic future solution, a realistic view emphasizes two concrete opportunities: (i) postassembly installation or exchange of targeting ligands and imaging probes on nanoparticle surfaces under mild, bio‐orthogonal conditions and (ii) site‐specific activation of prodrugs or caged agents at the tumor, using reactions that proceed rapidly and selectively in the presence of endogenous functional groups [9, 77].

To move these approaches toward breast cancer therapy, several practical challenges must be addressed, including the pharmacokinetics and potential toxicity of the reactive partners, the stability of the modified nanoparticles in complex biological fluids, and the scalability and reproducibility of click‐based manufacturing processes [78]. Carefully designed in vivo studies that quantify reaction efficiency, off‐target modification, and long‐term safety in relevant tumor models will be essential before biorthogonal chemistry can be considered a realistic adjunct to clinically oriented nanoparticle formulations

### 5.7. AI and Machine Learning Integration

In nanomedicine, AI and machine learning are increasingly investigated as tools to support, rather than replace, experimental design. Realistic short‐term applications for breast cancer include using AI‐assisted models to prioritize nanoparticle formulations based on physicochemical descriptors, to predict tumor versus normal tissue distribution from preclinical imaging datasets, and to identify patient subgroups most likely to benefit from nanocarrier‐based regimens [78]. These models can help to narrow the space of experimental design and generate testable hypotheses, but their ultimate utility depends on the quality and diversity of the underlying data and prospective validation.

Future work should focus on integrating model development with iterative experimental cycles, transparent reporting of model performance metrics, and incorporation of AI‐based predictions into the design of preclinical and early phase clinical studies [3] to ensure that AI‐guided nanoparticle design contributes to clinical translation. Within this evidence‐based framework, AI and machine learning are positioned as pragmatic decision support tools to expedite optimization of nanoparticle formulations and trial designs rather than as stand‐alone transformative technologies.

### 5.8. Biodegradable and Biocompatible Nanoparticle Materials

Investigation of new biodegradable and biocompatible nanoparticle‐making materials could help with long‐term toxicity concerns. The creation of nanoparticles involving materials that are safely metabolized or excreted by the body provides safer use, with high safety and low long‐term toxicity. This is evidenced using nanoparticles made from natural polymers (such as chitosan or alginate), which will break down in the body safely (e.g., reduced side effects and enhanced safety are reported) [44].

### 5.9. Advanced Imaging and Tracking

Integration of advanced imaging and tracking capabilities into nanoparticles allows for real‐time monitoring of drug delivery and drug therapy progress. Nanoparticles with improved imaging (e.g., with MRI, PET, and fluorescence) enhance treatment efficiency. This makes the treatment of such an issue possible. Dual‐modality nanoparticles showing simultaneous MRI and fluorescence imaging to enable tracking of delivery and distribution of tumors are an example of such a property.

### 5.10. Microenvironment‐Responsive Nanoparticles

Forming nanoparticles that respond to different conditions within the tumor microenvironment, such as hypoxia or increased extracellular matrix elements, facilitates drug targeting and release. To this end, the synthesis of nanoparticles that can respond to individual microenvironmental factors in tumors enables better controlled targeting and drug release. Nanoscale strategies have recently been used in clinical practice to target therapies to specific tumor sites and enable drug release control. This is achieved through nanoparticles that induce the release of drugs under hypoxic conditions, and, for instance, if specific enzymes are present in the tumor stroma [79].

In brief, the emerging approaches mentioned above show a mixture of convergence and divergence in the field. There is a strong consensus that stimuli‐responsive designs, combination nanotherapies, and integration with immunotherapy and advanced imaging have the potential to improve therapeutic precision greatly. However, studies differ in how convincingly they demonstrate added value beyond existing nanoparticle formulations or standard treatments [65]. The conflicting findings often relate to differences in tumor models, nanoparticle characterization, dosing schedules, and outcome measures, which make direct comparison problematic [19, 61]. To push the field forward, future work should focus on harmonized characterization protocols, clinically relevant breast cancer models (including metastatic and resistant disease), and comparative studies that directly test whether complex, next‐generation platforms provide meaningful advantages over simpler, clinically validated systems [3]. Such prioritization will help to separate high‐impact concepts with actual translational potential from attractive mechanistic designs that are unlikely to be feasible in routine oncology practice.

### 5.11. Clinical Translation Challenges, Toxicity, and Regulatory Issues

Despite significant preclinical advances in the use of nanoparticles for breast cancer therapy, clinical translation has been more modest than expected. Large‐scale studies of cancer nanomedicines reveal that although Phase I studies report high rates of acceptable safety and pharmacokinetics, many candidates do not continue to later phases, with overall Phase III success rates reported in the range of 10%–15%, and most discontinued products are discontinued due to lack of efficacy or safety concerns rather than lack of mechanistic rationale [8, 61]. In the case of breast cancer, the approved nanomedicines (liposomal and albumin‐bound formulations) have shown, in several trials, mainly benefits in improved tolerability and altered pharmacokinetics [3, 64]. Gains in progression‐free or overall survival in comparison to conventional chemotherapeutics have often been modest, indicating the need for realistic expectations on the clinical impact.

Toxicity profiles of systems with nanoparticles also continue to be a major hurdle. Depending on composition, size, charge, and surface chemistry, nanoparticles may cause dose‐dependent cytotoxicity, oxidative stress, and genotoxicity in normal tissues such as the liver, spleen, and kidney, in addition to their intended effects on tumor cells [80]. Off‐target accumulation in the reticuloendothelial system and prolonged retention can cause chronic inflammation, complement activation‐related pseudoallergy, and organ dysfunction, and these effects may not be fully captured in short‐term preclinical studies [81]. Therefore, comprehensive nanotoxicology testing, including standardized in vitro, ex vivo, and in vivo assays for cytotoxicity, immunotoxicity, and genotoxicity, is now considered essential before the clinical translation of new nanoformulations [80, 82].

Interactions with the immune system add further complexity to the clinical performance of cancer nanomedicines. Many nanosystems are quickly opsonized by plasma proteins and cleared by macrophages of the mononuclear phagocyte system, resulting in reduced tumor exposure, interpatient variability in pharmacokinetics, and in some cases accelerated blood clearance upon repeated administration [83, 84]. Furthermore, nanoparticles can stimulate or inhibit immunity: They can enhance the production of inflammatory cytokines, complement activation, and infusion reactions, but they can also impair phagocytic function or alter the tumor immune microenvironment in ways that may counteract antitumor immunity [16]. These complex immune interactions help to explain why carriers that appear advantageous in rodent tumor models do not always translate into better clinical outcomes in breast cancer patients.

Regulatory and manufacturing hurdles also hinder the bench‐to‐bedside transition of nanoparticle‐based therapies. Existing guidelines have been largely developed for small molecules and biologics, and there is still limited harmonization of requirements specific to nanomedicines on physicochemical characterization, batch‐to‐batch reproducibility, long‐term stability, and in vivo behavior [61, 81]. Scaling up from laboratory synthesis to GMP‐compliant, large‐scale production while maintaining tight control over particle size, polydispersity, surface chemistry, and drug loading remains technically demanding and expensive, which can hinder commercialization even for conceptually promising platforms [8, 61]. Regulators have become more demanding about the provision of solid evidence that new nanoformulations offer clear clinical or safety benefits compared with optimized standard‐of‐care regimens, raising the bar for approval even higher.

Together, these problems underscore that the translation of nanoparticle‐based breast cancer treatments is limited not only by scientific and technical challenges but also by toxicity concerns, immune clearance, variable clinical performance, and a complex regulatory environment. Future development will require the integration of translational considerations early in nanoparticle design, disease‐relevant models, immune and safety endpoints, and trial designs that can detect not only improvements in response but also meaningful reductions in long‐term toxicity and treatment burden for patients [3, 8, 19].

## 6. Conclusion

The accumulated evidence on nanoparticle‐based therapies for breast cancer indicates a field that has clearly moved beyond proof of concept, yet has not fully delivered the transformative clinical impact often anticipated. Preclinical and early clinical studies consistently show that liposomal, polymeric, metallic, magnetic, and other engineered nanoparticles can improve drug solubility, tumor accumulation, and therapeutic index compared with conventional formulations. However, these gains remain uneven and highly context‐dependent rather than universally applicable. In particular, the benefits observed to date tend to be modest in survival terms and are often offset by issues of complexity, cost, and uncertain long‐term safety.

Taken together, current data support the view that nanoparticle platforms should be regarded as enabling technologies that refine and personalize existing breast cancer treatments, rather than as stand‐alone replacements for surgery, radiotherapy, endocrine therapy, or systemic chemotherapy. Their greatest value presently lies in specific, well‐defined clinical niches—such as dose intensification with manageable toxicity, overcoming selected mechanisms of MDR, improving drug penetration in poorly perfused lesions, or codelivering synergistic drug or imaging combinations—where conventional regimens alone are clearly inadequate.

At the same time, substantial translational barriers continue to limit broader clinical adoption. Tumor and patient heterogeneity, variability of the EPR effect, incomplete understanding of nano‐biointeractions, challenges in large‐scale reproducible manufacturing, regulatory uncertainty, and cost‐effectiveness considerations all constrain the generalizability of promising experimental results. Without systematic efforts to address these issues, the field risks generating increasingly sophisticated nanoparticle designs that are difficult to implement beyond specialized centers or narrow patient subgroups.

Looking ahead, the most urgent priorities are clear. First, rigorously designed, biomarker‐driven clinical trials are required to identify which molecular subtypes of breast cancer and which patient populations truly benefit from specific nanoparticle platforms. Second, harmonized standards for physicochemical characterization, in vivo behavior, safety assessment, and reporting must be adopted to allow meaningful comparison between studies and to accelerate regulatory evaluation. Third, deeper integration of stimulus‐responsive and biomimetic designs with immunotherapy, targeted agents, and microenvironment‐modulating strategies is needed to convert pharmacokinetic advantages into durable, clinically relevant responses. Finally, AI‐assisted formulation design and the development of biodegradable, immunologically acceptable materials, coupled with scalable manufacturing, will be essential for moving from bespoke prototypes to broadly accessible products.

In our view, the field of nanoparticle‐based breast cancer therapy currently stands at an inflection point: no longer exploratory, but not yet standard of care. If the scientific, clinical, and regulatory challenges outlined above are addressed in a coordinated manner, nanoparticle‐based strategies are poised over the coming decade to evolve from adjunctive tools into integral components of precision breast cancer management, improving both outcomes and quality of life for carefully selected patient groups.

## Author Contributions

All authors contributed equally to this work.

## Funding

No funding was received for this manuscript.

## Disclosure

All authors agree to publish this article in the journal.

## Ethics Statement

The authors have nothing to report.

## Conflicts of Interest

The authors declare no conflicts of interest.

## Data Availability

The data that support the findings of this study are available from the corresponding authors upon reasonable request.
